# Transanal total mesorectal excision: short- and long-term results of the first hundred cases of a certified colorectal cancer center in Germany

**DOI:** 10.1007/s00464-021-08384-3

**Published:** 2021-03-01

**Authors:** Vinzenz Völkel, Sabine Schatz, Teresa Draeger, Michael Gerken, Monika Klinkhammer-Schalke, Alois Fürst

**Affiliations:** 1 Tumorzentrum Regensburg - Institut für Qualitätssicherung und Versorgungsforschung der Universität Regensburg, Am BioPark 9, 93053 Regensburg, Germany; 2 Caritas Krankenhaus St. Josef Regensburg, Klinik für Allgemein-, Viszeral, Thoraxchirurgie und Adipositasmedizin, Landshuter Str. 65, 93053 Regensburg, Germany; 3 Caritas Krankenhaus St. Josef Regensburg, Klinik für Allgemeine Innere Medizin, Gastroenterologie und Kardiologie, Landshuter Str. 65, 93053 Regensburg, Germany

**Keywords:** Minimal-invasive surgery, NOTES—natural orifice transluminal endoscopic surgery, TaTME, Low rectal carcinoma, Clinical implementation

## Abstract

**Background:**

Since 2010, laparoscopic transanal total mesorectal excision (TaTME) has been increasingly used for low and very low rectal cancer. It is supposed to improve visibility and access to the dissection planes in the pelvis. This study reports on short- and long-term outcomes of the first 100 consecutive patients treated with TaTME in a certified German colorectal cancer center.

**Patients and methods:**

Data were derived from digital patient files and official cancer registry reports for patients with TaTME tumor surgery between July 2014 and January 2020. The primary outcome was the 3-year local recurrence rate and local recurrence-free survival (LRFS). Secondary endpoints included overall survival (OAS), disease-free survival (DFS), operation time, completeness of local tumor resection, lymph node resection, and postoperative complications. The Kaplan–Meier method was employed for the survival analyses; competing risks were considered in the time-to-event analysis.

**Results:**

During the observation period, the average annual operation time decreased from 272 to 178 min. Complete local tumor resection was achieved in 97% of the procedures. Major postoperative complications (Clavien–Dindo 3–4) occurred in 11% of the cases. At a median follow-up time of 2.7 years, three patients had suffered from a local recurrence. Considering competing risks, this corresponds to a 3-year cumulative incidence rate for local recurrence of 2.2% and a 3-year LRFS of 81.9%. 3-year OAS was 82.9%, and 3-year DFS was 75.7%.

**Conclusion:**

TaTME is associated with favorable short and long-term outcomes. Since it is technically demanding, structured training programs and more research on the topic are indispensable.

## Background

In the past years, the laparoscopic approach has become the new standard even for tumor resections in the lower rectum. According to a variety of studies, it is associated with better short-term and at least equivalent oncologic long-term outcomes compared to the conventional open approach [1, 2]. The so-called transanal total mesorectal excision (TaTME) technique represents the latest development in the field of minimally invasive surgery for low and very low rectum tumors. Following this approach, tumor resection is achieved by a combination of laparoscopic abdominal and endoscopic transanal preparation. TaTME is supposed to improve visibility and access to the dissection planes in the pelvis leading to a greater degree of radical resection, lower rates of anastomotic leakage, more sphincter-saving procedures, and a better preservation of the urogenital function [3, 4]. It was first performed in 2009 and described in 2010 by Sylla et al. [5]. In the meantime, the technique has been implemented in more than 300 centers worldwide [6]. As of today, the evidence base for the TaTME technique mainly consists of observational studies with heterogeneous results. While many publications, mainly by expert centers on the new technique, reported favorable outcomes [7–14], the TaTME registry, representing more than 100 centers from all over the world voluntarily entering data, recorded an anastomotic failure rate of 15.6% among 1594 patients and a circumferential resection margin positive rate of 4.4% [15]. According to a recent publication based on Norwegian cancer registry data, the local recurrence rate after TaTME was 9.5%, as opposed to 3.4% after the conventional approach [16, 17]. This caused a vivid discussion about possible deficits in the Norwegian TaTME training program [18]. Moreover, there were doubts concerning the extraordinarily good results after conventional procedures. Currently, the COLOR III [19] and the GRECCAR 11 [20] studies, the first large randomized trials on the topic, are under progress, but recruitment is not finished yet. The present study reports on short- and oncologic long-term outcomes of the first patients treated with TaTME in one of currently four German hospitals participating in the COLOR III study.

## Patients and methods

This retrospective cohort study was conducted in a certified German colorectal cancer center with an average annual volume of 110 primary colorectal cancer cases during the last three years. The surgical team completed several TaTME training courses according to the Consensus on structured training curriculum for transanal total mesorectal excision [21] before the first patient was treated in this technique. This paper aims to describe the treatment and outcomes of the first 100 consecutive patients who received a laparoscopic resection of their rectum carcinoma in TaTME technique between July 2014 and January 2020. Prior to surgery, every patient’s case was presented to an interdisciplinary tumor board proposing the optimal therapy pathway. Pre- and postoperative radiochemotherapy was performed according to the evidence-based recommendations of the German S3-treatment guideline [22]. For all surgical procedures, the EinsteinVision® 3D-imaging system, the GelPOINT® transanal access platform (AppliedR), and CONMED's AirSeal® System were used. After installation of the single-port system, a circular pursestring suture was placed before the dissection took place. In some cases of intersphincteric resections, the dissection was performed first followed by the pursestring suture. A perineal washout was performed before and after the placement of the pursestring suture routinely. In the majority of patients, a stapled anastomosis, in case of intersphincteric resections, a hand-sewn anastomosis was conducted.

Information on each patient includes personal features like age or body mass index (BMI), tumor characteristics like clinical and pathological TNM/ Union for International Cancer Control (UICC) stage, details on (additional) treatment procedures like (neo)adjuvant radiochemotherapy, and clinical outcomes like postoperative complications. Information was extracted directly from the hospital’s digital patient files including all kind of medical documents like discharge documents, (external) diagnostic reports, surgical reports, anesthesiologic reports, pathological reports, and notes on subsequent follow-up visits. Moreover, a data complement to the official clinical cancer registry database of the region, maintained by Tumorzentrum Regensburg/ Institute for Quality Management and Health Services Research of University of Regensburg (TUZR), was conducted to obtain information about postoperative recurrence events. By law, TUZR longitudinally collects all available information on every tumor patient registered within the wider catchment area of the analyzed hospital. The actual life status was retrieved directly from the local registration offices. In compliance with German data protection laws, all concerned patients had to give their written consent to the anonymized use of their data.

Primary endpoint was the local recurrence rate together with the local recurrence-free survival (LRFS). Secondary endpoints included overall survival (OAS), disease-free survival (DFS, defined as the length of time after primary treatment without any signs or symptoms of the analyzed rectal cancer), operation time, completeness of local tumor resection according to the Mercury- and the R-classification, the number of harvested lymph nodes, and postoperative complications according to the Clavien–Dindo classification [23, 24]. While Clavien–Dindo 1 and 2 describe any deviation from the normal postoperative course which can be dealt with by pharmaceutical means, Clavien–Dindo 3 indicates complications requiring surgical, endoscopic, and radiological interventions; this, for example, includes postoperative hernia or superficial abscess. Potentially life-threatening complications like, e.g., peritonitis are classified as Clavien–Dindo 4.

Descriptive statistics include categorization in case of nominal variables; for scalar variables, the median and the mean together with the standard deviation were calculated. In all Kaplan–Meier survival analyses, the date of surgery served as starting point. Local recurrence, metachronous distant metastasis, and death were considered as events. Recurrences within three months after surgery were regarded as early events. If a patient was consecutively affected by two or more events, only the first was treated as endpoint for the correspondent analyses. To obtain an accurate local recurrence rate in the presence of death and distant metastasis as competing risks, the corresponding cumulative incidence rate was calculated. To give the reader a comprehensive overview on all observed recurrence events, a table with relevant information on each of the affected cases is provided. It describes type and location of the recurrent tumor tissue together with detailed information on the originally resected primary tumor and the preceding therapy pathway.

This study is an observational retrospective trial not involving any experiments with patients or animals; therefore, no IRB approval was necessary. The findings of this survey are presented in strict compliance with the Strengthening the Reporting of Observational studies in Epidemiology (STROBE) statement [25]. For the statistical analyses, IBM SPSS 26 (IBM Corp., SPSS for Windows, Armonk, NY, USA), R version 3.5.1 (R Foundation for Statistical Computing, Vienna, Austria; http://www.R-project.org/), and the R package “cmprsk” were used.

## Results

Between July 2014 and January 2020, 100 patients received an oncologic rectum resection in TaTME technique. In the first two years, twelve patients were treated, between 2016 and 2018, the annual case load was constantly above 20, while in 2019, 14 TaTME procedures were performed. No intraoperative conversion to an abdominoperineal resection occurred, but once, a TaTME was intraoperatively transformed into a classical abdominal laparoscopic low anterior resection. This case was excluded from further analyses.

Of the included patients, 65 were male, and 35 were female. The median age at the time of surgery was 62.9 years, the mean 63.1 years (standard deviation, sd, 11.1) with a minimum of 36.6 and a maximum of 84.8 years. The patients’ BMI ranged between 18.7 and 43.1 kg/m^2^ with a median of 26.3 kg/m^2^ and an average of 27.1 kg/m^2^ (sd 4.8), indicating moderate overweight. Seventy patients were classified as American Society of Anesthesiologists Physical Status System (ASA) 1 or 2, 29 as ASA 3, and only one as ASA 4. (cf. Table 1).

**Table 1:** Patients’ characteristics (BMI = body mass index, ASA = American Society of Anesthesiologists Physical Status System)

		n/%
Sex	male	65
	female	35
Age	< 50	12
	50–59	29
	60–69	31
	70–79	22
	≥ 80	6
BMI	< 25	37
	25–30	37
	30–40	23
	> 40	3
ASA	1/2	70
	3	29
	4	1

The resected tumors were located between 4 and 11 cm from the anocutaneous line with an average distance of 7.3 cm (sd 1.8). 88 of the resected specimens were classified as Mercury 1, nine as Mercury 2 and in three cases, no Mercury classification is available. At diagnosis, the majority of patients (*n* = 80) were classified as UICC stage III, six patients already had distant metastases (UICC IV). After surgery (and in 85 cases after preceding neoadjuvant treatment), 33 tumors were diagnosed as UICC I, 15 as UICC II, and 22 as UICC III. In 21 patients, no tumor tissue had been detected in the resected specimens anymore, while the number of UICC IV cases had increased to 9. (cf. Table 2).

**Table 2:** Tumor characteristics (UICC = Union for International Cancer Control staging system)

		n/%
Tumor height	4-5 cm	24
	6-8 cm	49
	9-11 cm	27
Mercury classification	1	88
	2	9
	unknown	3
cUICC	I	7
	II	7
	III	80
	IV	6
cT	1	4
	2	12
	3	81
	4	3
cN	0	14
	1	77
	2	9
cM	0	94
	1	6
(y)pUICC	0/is	21
	I	33
	II	15
	III	22
	IV	9
(y)pT	0	22
	1	14
	2	25
	3	36
	4	3
(y)pN	0	72
	1	21
	2	7
(y)p/cM	0	91
	1	9

**Fig. 1:**
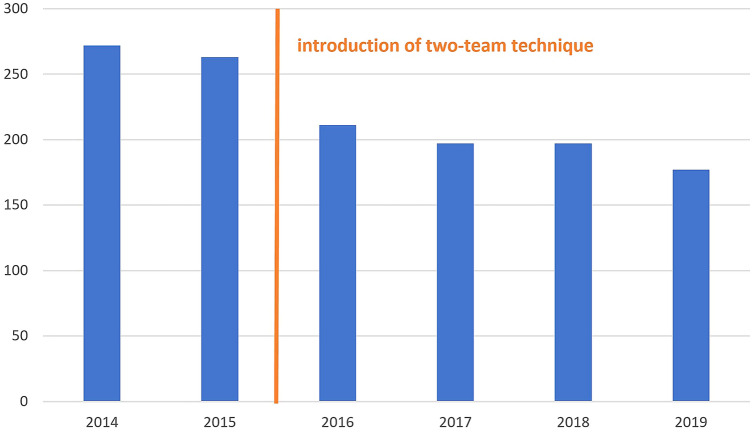
Annual means of the operation time in minutes

Before surgery, 85 patients obtained a neoadjuvant therapy and 65 received an additional adjuvant chemotherapy afterwards in compliance with the German guidelines. Seventy-three patients underwent a low anterior resection (LAR), in 27 cases an intersphincteric resection (ISR) was necessary (cf. Table 3). Ninety-nine patients obtained a protective ileostomy; in eight cases, this ileostomy was created prior to the tumor resection in a separate operation. One patient received a permanent colostomy. The operation time ranged between 74 and 380 min. The annual means for the operation time decreased from 272 min in 2014 to 211 min in 2016. At this time, the so-called two-team technique involving simultaneous preparation from abdominal and transanal was introduced. Thereafter, the operation time could be further reduced to 178 min in 2019 (cf. Fig. 1). The time from surgery to hospital discharge was 12.4 days (sd 10.8) on average with a wide range of 6 to 90 days.

**Table 3:** Treatment (*LAR* low anterior resection, *ISR*  intersphincteric resection, *CRT*  chemoradiotherapy, *RT*  radiotherapy, *CT* chemotherapy)

		n/%
Neoadjuvant therapy	CRT	76
	RT	8
	CT	1
	no	15
Adjuvant therapy	CT	65
	no	34
	unknown	1
Type of surgery	LAR	73
	ISR	27
Ostomy	protective Ileostomy	99
	permanent colostomy	1

No intraoperative complications have been documented for any of the patients. A complete resection of the primary tumor was achieved in 97 of 100 patients. Twelve or more lymph nodes were harvested and examined in 90 cases. Sixty-eight patients had no postoperative complications (Clavien–Dindo 0) in the first 30 days following surgery. In 21 patients, minor complications like intestinal hypomotility or irregular wound healing occurred. Eight patients had major (Clavien–Dindo 3) or potentially life-threatening (Clavien–Dindo 4) complications. Anastomotic leakage has been diagnosed in seven patients, and presacral abscess in two patients. In three cases, a major reoperation involving a re-opening of the abdominal cave was necessary (cf. Table 4).

**Table 4:** Quality indicators, postoperative complications

		n/%
Local residual tumor	R_local_ 0	97
	R_local_ 1	3
Harvested lymph nodes	< 12	10
	≥ 12	90
Postoperative complications (within 30 days)	Clavien–Dindo 0	68
	Clavien–Dindo 1/2	21
	Clavien–Dindo 3	8
	Clavien–Dindo 4	3

	anastomotic leakage	7
	presacral abscess	2
	major reoperation*	3

^*^Involving re-opening of the abdominal cave

The median (mean) follow-up time of the patient cohort was 2.7 (2.6) years, ranging between 2.2 months and 5.8 years. During that time period, nine patients died, twelve had a metachronous distant metastasis as first event of which two occurred in the first three months after surgery, and three developed a primary local recurrence. This corresponds to a 3-year cumulative incidence rate of 2.2% for the endpoint local recurrence in the presence of death and metachronous distant metastasis as competing events. Table 5 provides further details on recurrent cases. None of the patients with a local recurrence event had received pre- or postoperative radiochemotherapy of the primary tumor and good tumor regression (Dworak 4 or higher) was only achieved in two out of twelve patients with metachronous distant metastases.

**Table 5:** Patients with local (LR) or metachronous distant metastasis recurrence (DM), LAR = low anterior resection, ISR = intersphincteric resection, CRT = chemoradiotherapy, CT = chemotherapy, LN = lymph nodes

Type of event	time to event	Location	cTNM of primary tumor	pTNM of primary tumor	Dworak	Procedure	R_local_	Perioperative treatment	Postoperative complications
LR	4.4 months	10 cm a.a	cT3cN1cM0	pT3pN2cM0	–	LAR	R0	–	Paralytic Ileus
LR	12.4 months	8 cm a.a	cT2cN0cM0	pT3pN1cM0	–	LAR	R0	–	–
LR	36.1 months	10 cm a.a	cT2cN0cM0	pN2pN0cM0	–	LAR	R0	–	–
Early DM (hep.)	1.0 months	5 cm a.a	cT3cN1cM0	ypT3ypN1cM0	1	ISR	R1	Neoadjuvant CRT no adj. therapy	Presacral abscess
Early DM (hep.)	1.5 months	9 cm a.a	cT3cN1cM0	ypT3ypN2cM0	2	LAR	R0	Neoadjuvant CRT adjuvant CT	–
DM (pul.)	6.9 months	10 cm a.a	cT3cN1cM0	ypT0ypN0cM0	4	LAR	R0	Neoadjuvant CRT adjuvant CT	–
DM (pul.)	7.3 months	9 cm a.a	cT3cN1cM0	ypT3ypN0cM0	3	LAR	R0	Neoadjuvant CRT adjuvant CT	Acute kidney failure, prolonged wound healing
DM (pul.)	8.0 months	5 cm a.a	cT3cN1cM0	ypT3ypN0cM0	1	ISR	R0	Neoadjuvant CRT	Presacral abscess, anastomotic leakage, major reoperation
DM (hep.)	8.0 months	6 cm a.a	cT3cN2cM0	ypT3ypN2cM0	2	ISR	R0	Neoadjuvant CRT adjuvant CT	–
DM (pul., LN)	9.4 months	9 cm a.a	cT3cN1cM0	ypT3ypN1cM0	3	LAR	R0	Neoadjuvant CRT	Prolonged wound healing with VAC therapy
DM (pul.)	11.2 months	9 cm a.a	cT3cN1cM0	ypT3ypN1cM0	1	LAR	R0	Neoadjuvant CRT adjuvant CT	Prolonged wound healing
DM (hep.)	11.3 months	8 cm a.a	cT3cN1cM0	ypT3ypN1cM0	1	LAR	R0	Neoadjuvant RT adjuvant CT	–
DM (hep.)	17.9 months	10 cm a.a	cT3cN2,cM0	cT3cN0cM0	2	LAR	R0	Neoadjuvant CRT adjuvant CT	–
DM (hep.)	39.5 months	10 cm a.a	cT3cN1cM0	ypT2ypN0cM0	2	LAR	R0	Neoadjuvant CRT adjuvant CT	–
DM (pul.)	46.8 months	5 cm a.a	cT3cN1cM0	ypT0ypN0cM0	4	ISR	R0	Neoadjuvant CRT	Macrohematuria

**Fig. 2:**
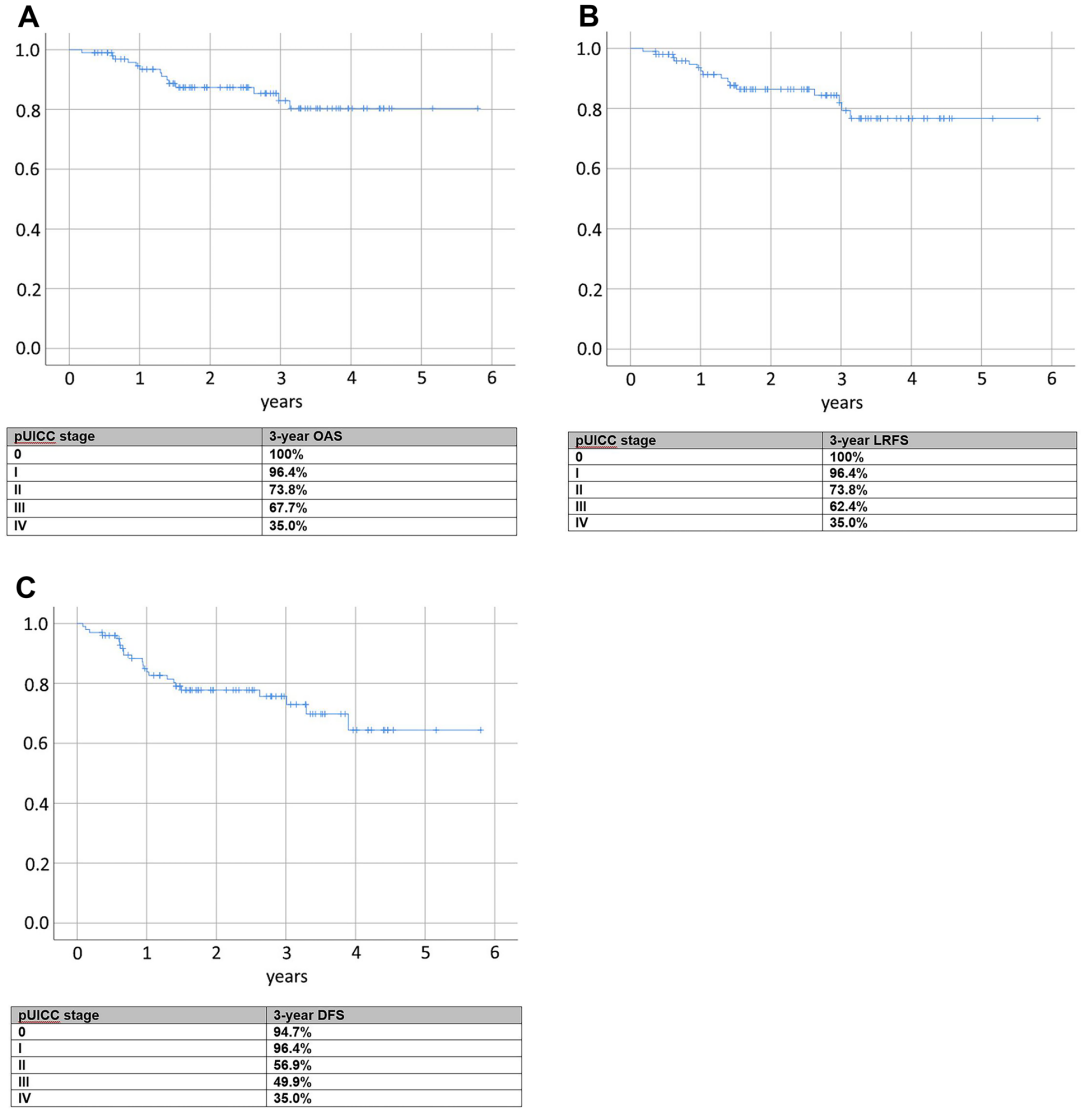
**A** Kaplan–Meier curve for overall survival (OAS). **B** Kaplan–Meier curve for local recurrence-free survival (LRFS). **C** Kaplan–Meier curve for disease-free survival (DFS)

The overall Kaplan–Meier 3-year survival rate is 82.9%, ranging between 100% for patients with a postoperative UICC stage 0 and 35.0% for UICC IV patients (cf. Fig. 2A).The 3-year Kaplan–Meier local recurrence-free survival rate is 81.9%. For patients with a postoperative UICC stage 0, it is 100% and decreases to 35.0% for UICC IV patients (cf. Fig. 2B). The 3-year Kaplan–Meier disease-free survival rate is 75.7% and ranges between 94.7% for patients with a postoperative UICC stage 0 and 35.0% for UICC IV patients (cf. Fig. 2C).

## Discussion

This study documents the successful implementation of the new TaTME technique in the daily clinical practice of a certified colorectal cancer center in Germany. Without doubt, TaTME is associated with a learning curve, reflected by a 35% decline of the average annual operation time during the observation period; between the second (2015) and the third year (2016), the largest drop can be observed. This most likely has to be attributed to the implementation of the so-called two-team technique with simultaneous minimal-invasive preparation from abdominal and transanal, leading to a “rendezvous” in the course of the operation. With a mean operative time of 178 min, the analyzed hospital drew level with the 166 min reported by Lacy et al. as an average for their first 140 cases [8].

Even in the international comparison, the observed postoperative results are extraordinarily promising: At the end of 2019, Hol et al. published their results of 159 consecutive patients treated with TaTME in two referral centers in the Netherlands [26]. They reported a postoperative complication rate of 52.2% (Clavien–Dindo 1–4) as opposed to 32.0% in our study. Focusing on presacral abscesses (2.0% vs. Hol et al.: 8.8%) and reoperation (3.0% vs. Hol et al.: 22.6%), we found more favorable results, too. Concerning anastomotic leakage, our results (7.0%) are equivalent to the Dutch outcomes (6.3%) and considerably better than the 15.6% mentioned in the TaTME registry report [15]. It has to be underlined that, apart from age (mean: 63.1 vs. Hol et al.: 66.9), our cohort shows less favorable patient and tumor characteristics then the Dutch cohort. The patients in our study have a higher average BMI and a higher share of more advanced tumor and nodal stages. Moreover, there are even patients who did not receive neoadjuvant or adjuvant treatment, although it would have been formally indicated. The rate of intersphincteric resections (27.0%) with a higher risk of incomplete resection was higher in our cohort than in the Dutch cohort (16.4%). Nevertheless, with 3.0%, the rate of incomplete local tumor resections (R1) was also a bit higher in our cohort than in the cohort of Hol et al. (0.6%).

The minimum and mean follow-up time of the Dutch publication is 36 months and 54.8 months, respectively; this provides more favorable statistical conditions for survival and time-to event analyses then the mean follow-up time of 31.3 months observed in our study. However, although Hol et al. postulate a 3-year local recurrence rate of 2%, they reported six cases of local recurrence within 30 months after surgery in their study. As a matter of fact, this is equivalent to a 3-year local recurrence rate of at least 3.8% if censoring is not taken into account. In our study, we observed three recurrence events among 100 patients during a median follow-up time of 2.7 years. If competing risks are factored in, the corresponding 3-year local recurrence rate ranges slightly above 2%. Thus, the outcomes of the German hospital analyzed by this study are considerably better than the long-term results reported by Lacy at all for their first 140 cases. They observed a local recurrence rate of 2.3% at a mean follow-up time of fifteen months [8].

Concerning our study, one can see that none of the patients with a local recurrence did receive neoadjuvant and/or adjuvant treatment, although it would have been formally indicated by the guidelines. Reasons for this were refusal by the patient or a negative vote by the interdisciplinary tumor board due to a weak overall health status. Therefore, it remains unclear whether the observed local recurrence events can be exclusively attributed to the surgical technique. Notwithstanding this, it becomes apparent that the local recurrence rates both in our study and the study from Hol et al. are considerably lower than the recently published 10% observed in another Dutch implementation cohort [6] and the 9.5% reported by the Norwegian cancer registry [16, 17]. The 3-year overall survival rates of both studies are comparable (Hol et al. 83.6% vs. 82.9% in our study).

Although the results of the present study are quite promising, some limitations have to be considered: We cannot exclude that there is some bias involved concerning the selection of TaTME patients. However, this is something our study has in common with all observational studies on the topic. Moreover, it has to be taken into account that the hospital our study was conducted in is a colorectal cancer center which received its certification already in 2009; therefore, it features profound experience in the application of colorectal cancer surgery. It regularly participates in national and international research projects, supports the wider dissemination of TaTME by providing structured training programs to novices in the new technique, and applies advanced technical gear like three-dimensional imaging. Moreover, all principal surgeons have considerable experience with minimally invasive techniques.

It has been shown that outcomes improve quickly as experience with a new surgical technique increases [27]. Admittedly, a less steep learning curve than observed in this study has to be expected in hospitals with lower standards or less experience in the TaTME technique. A systematic review by Deijen et al. revealed considerable differences between high- and low-volume centers performing TaTME: According to them, the TME-quality was more often assessed as “complete,” and the major complication rate and the local recurrence rate were lower in high-volume centers [28]. Similar to other procedures in colorectal surgery [29, 30], certain annual minimum caseloads and the adherence to defined quality standards seem to be an indispensable pre-requisite to achieve results like presented in this study. This supports the suggestion by the TaTME consensus group that a minimum of 14 procedures per year have to be performed at a site in order to achieve optimal quality of the procedure [31]. The mentioned results reported by the international TaTME registry [15] are based on an average of less than 6 cases per hospital and year, which is an indicator for a low level of experience. The even worse results reported by the Norwegian cancer registry [16, 17] might also be attributed to a lack of familiarity with the new technique.

TaTME is definitely technically demanding. Therefore, careful patient selection and structured training programs like they have been enrolled in the Netherlands are a key element for its further successful implementation [32, 33]. Whether or not the TaTME technique provides added oncologic value to the conventional technique requires results from the currently ongoing international multicenter trials COLOR III and GRECCAR 11. More studies comparing TaTME to the classical laparoscopic and maybe also robotic-assisted techniques are obligatory.

## Conclusion

TaTME is a technically demanding surgical approach requiring adequate training programs. Given sufficient experience on site, TaTME is associated with favorable short- and long-term outcomes. The local recurrence rate in this study proofed to be relatively low compared to other studies on the topic.
